# Epidural Catheter- and Port-Related Infections in Pain Medicine: A Cohort Study from a Chronic Pain Center with Long-Term Catheterization

**DOI:** 10.3390/pathogens15040349

**Published:** 2026-03-25

**Authors:** Mesut Bakır, Esra Bayburtluoğlu, Bedri İlcan, Kaan Yavuz, Nureddin Teker, Şebnem Rumeli

**Affiliations:** Division of Pain Medicine, Department of Anesthesiology and Reanimation, Mersin University, Mersin 33343, Türkiye

**Keywords:** epidural catheter, epidural port, catheter-related infection, pain medicine, catheter duration

## Abstract

Epidural catheters and epidural port systems are widely used in interventional pain management; however, infectious complications remain an important safety concern, particularly during prolonged catheterization. This retrospective real-world cohort study evaluated the incidence, microbiological characteristics, and time-dependent risk factors of epidural catheter- and port-related infections in a tertiary pain clinic. A total of 352 patients were included after predefined exclusion criteria were applied. Infection was defined based on clinical findings and microbiological confirmation. The overall infection rate was 10.5% (37/352). Catheter duration was significantly longer in patients who developed infection and remained an independent predictor in multivariable logistic regression analysis (odds ratio per day: 1.12; 95% CI: 1.06–1.19; *p* = 0.002). Receiver operating characteristic analysis demonstrated moderate discriminatory ability of catheter duration for predicting infection (AUC = 0.68), with an optimal cut-off of 7 days. The most frequently isolated pathogens were methicillin-resistant coagulase-negative staphylococci. No cases of meningitis or sepsis occurred, and all infections were successfully managed with antibiotic therapy. These findings emphasize the importance of duration-based risk assessment and careful follow-up strategies in patients requiring prolonged epidural analgesia.

## 1. Introduction

Epidural catheterization is a widely used technique in pain medicine for the management of cancer-related pain, postoperative pain, and other chronic pain conditions requiring prolonged analgesia [1]. Despite its established efficacy, epidural catheter use is associated with a risk of infectious complications, which may lead to significant morbidity if not recognized and managed promptly [2].

Infectious complications related to neuraxial techniques range from localized catheter-site infections to severe conditions such as epidural abscess or meningitis, although the latter are relatively rare in contemporary practice [3]. Large observational studies and narrative reviews have emphasized that the risk of infection is strongly influenced by procedural factors and catheter dwell time rather than the neuraxial technique itself [4].

Patients managed in pain clinics differ substantially from perioperative anesthesia populations, as they often require longer catheterization periods and repeated catheter access [5]. Previous studies suggest that infection rates in pain medicine settings may be higher than those reported after short-term perioperative epidural catheter use [6]. Prolonged catheter duration has consistently been identified as the most important modifiable risk factor for epidural catheter-related infection [7]. Registry analyses and systematic reviews have demonstrated a time-dependent increase in infection risk, particularly when catheterization exceeds several days [8].

Previous studies have suggested that the risk of catheter-related infection increases with prolonged catheterization, particularly beyond the first week of catheter use [7]. This increase in risk has been attributed to progressive bacterial colonization and biofilm formation on indwelling catheter surfaces [9]. Therefore, identifying clinically relevant time thresholds for infection risk remains an important objective in studies evaluating long-term epidural catheter use [9].

In recent years, epidural port systems and tunneled catheter techniques have been introduced to facilitate long-term neuraxial analgesia, especially in patients with cancer-related pain [10]. However, data regarding infection risk associated with epidural port systems remain limited, and existing evidence suggests that catheter duration rather than the access system itself may be the primary determinant of infection [11].

Microbiological studies have consistently identified skin-derived organisms, particularly methicillin-resistant coagulase-negative staphylococci, as the most common causative pathogens in catheter-related infections, highlighting the importance of aseptic technique and catheter maintenance [12,13]. Despite increasing awareness and updated infection control guidelines, real-world data focusing on epidural catheter and epidural port use in pain clinics remain scarce, particularly with regard to time-based risk assessment and clinically applicable cut-off values for catheter duration [2].

Therefore, the present study aimed to evaluate the incidence, risk factors, and microbiological characteristics of epidural catheter- and epidural port-related infections in a tertiary pain clinic, with a particular focus on catheter duration and the identification of a clinically meaningful time threshold associated with increased infection risk.

## 2. Materials and Methods

### 2.1. Study Design and Setting

This study was designed as a single-center, retrospective observational study conducted at the Pain Clinic of Mersin University Faculty of Medicine. Medical records of patients who underwent epidural catheter or epidural port placement for pain management between January 2002 and January 2025 were reviewed.

### 2.2. Ethical Approval and Trial Registration

The study protocol was approved by the Mersin University Clinical Research Ethics Committee (Approval No: 78017789/050.01.04/3257639; Date: 23 September 2025). The study was registered at ClinicalTrials.gov (Identifier: NCT07242560). Given the retrospective design and the use of anonymized data, the requirement for informed consent was waived by the ethics committee.

### 2.3. Study Population

A total of 386 patients who underwent epidural catheter or epidural port placement were initially screened. Patients were evaluated based on predefined inclusion and exclusion criteria.

#### 2.3.1. Inclusion Criteria

Patients were included in the study if they met all of the following criteria:

- Age ≥ 18 years;
- Epidural catheter or epidural port placement performed and followed up by the pain clinic;
- Complete medical records available, including catheter duration and infection status;
- Follow-up data sufficient to evaluate catheter- or port-related infectious complications.

#### 2.3.2. Exclusion Criteria

Patients were excluded from the study if they met any of the following criteria:

- Patients with systemic infection or sepsis at the time of catheter placement were excluded to minimize potential confounding factors related to critical illness and to allow a more specific evaluation of catheter-related infection risk in the pain clinic population.
- Admission to the intensive care unit during catheterization or follow-up.
- Incomplete or missing clinical records relevant to the study outcomes.

Based on these criteria, 34 patients were excluded from the analysis. The final study population consisted of 352 patients, all of whom had complete records and were regularly followed up by the pain clinic.

### 2.4. Data Collection

Demographic characteristics, clinical diagnoses, procedural details, and microbiological data were extracted from institutional medical records. Collected variables included catheter or port type, insertion level, indication for catheter placement, duration of catheterization, infection status, and microbiological culture results when available.

Catheter-related infection was defined as the presence of documented local clinical signs at the catheter insertion site (such as erythema, purulent discharge, tenderness, or local inflammation), with microbiological confirmation when bacterial growth was detected. Cases considered by the microbiology department to represent catheter colonization without accompanying clinical findings were excluded from the analysis.

When a systemic infection was clinically suspected, additional diagnostic investigations, including blood cultures, were obtained according to institutional infection management protocols. Due to the retrospective design of the study, blood culture data were not consistently available for all cases and were therefore not included in the formal analysis.

#### Microbiological Sampling and Culture Procedure

In our clinic, microbiological sampling from epidural catheters and epidural port systems is performed as part of routine clinical practice in cases with suspected infection or at the time of catheter or port removal. In accordance with recommendations from the Department of Microbiology, culture samples were obtained by aseptically cutting the distal tip of the epidural catheter or port system. In addition, the in-line filter used during catheterization was flushed with sterile distilled water, and the effluent was collected for microbiological analysis.

All samples were promptly transported to the microbiology laboratory in sterile tubes. Catheter specimens were first inoculated into thioglycolate broth medium. After vortex agitation, the samples were plated onto 5% sheep blood agar and eosin methylene blue (EMB) agar. In addition, Gram-stained and Giemsa-stained smears were prepared and examined microscopically. Cultures were incubated at 36 °C for 18–48 h and evaluated for microbial growth. At the end of the incubation period, samples showing turbidity in thioglycolate broth were subcultured onto both media for further evaluation.

Microorganisms isolated from positive cultures were identified using standard microbiological methods. Identification was based on colony morphology, Gram staining characteristics, and conventional biochemical testing. Antimicrobial susceptibility testing was performed according to routine laboratory protocols in accordance with current clinical microbiology standards. This definition was applied in accordance with routine clinical practice and is consistent with the principles outlined in contemporary infection control guidelines, including recent ASRA Pain Medicine recommendations [2].

When a catheter- or port-related infection was clinically suspected, the epidural catheter or port system was removed whenever clinically appropriate. Empirical antibiotic therapy was initiated according to institutional infection management protocols and subsequently adjusted based on microbiological culture results and antimicrobial susceptibility testing. Patients were followed by the pain clinic team until clinical resolution of the infection was documented.

### 2.5. Outcome Measures

The primary outcome was the development of epidural catheter- or port-related infection. Secondary outcomes included identification of factors associated with infection development and characterization of the causative microorganisms.

### 2.6. Statistical Analysis

Continuous variables were assessed for normality and are presented as mean ± standard deviation or median (minimum–maximum), as appropriate. Categorical variables are expressed as counts and percentages.

Comparisons between patients with and without infection were performed using the Mann–Whitney U test for continuous variables and the chi-square or Fisher’s exact test for categorical variables, as appropriate. Variables demonstrating potential association with infection were included in a multivariable logistic regression model to identify independent risk factors. Results are reported as odds ratios (ORs) with 95% confidence intervals (CIs).

Receiver operating characteristic (ROC) curve analysis was conducted to evaluate the predictive value of catheter duration for infection development and to determine the optimal cut-off value. Because the exact timing of infection onset was not systematically recorded and the temporal relationship between symptom onset, diagnosis, and catheter removal could not be reliably determined in this retrospective dataset, time-to-event analysis using Cox regression was not feasible. Therefore, logistic regression analysis was used as a more appropriate approach to evaluate the association between catheter duration and infection development. A *p* value < 0.05 was considered statistically significant.

All statistical analyses were performed using SPSS software (version 22.0; IBM Corp., Armonk, NY, USA).

## 3. Results

### 3.1. Patient and Procedural Characteristics

A total of 352 patients who underwent epidural catheter or epidural port placement were included in the final analysis. Epidural catheter- or port-related infection was observed in 37 patients, corresponding to an overall infection rate of 10.5%**.**

Patients were categorized according to the clinical indication for catheter placement into three groups: cancer-related pain, postoperative surgical pain, and other indications. The “other indications” category included various chronic pain conditions such as postherpetic neuralgia, complex regional pain syndrome, failed back surgery syndrome, and refractory neuropathic pain. The majority of patients belonged to the “other indications” group, followed by cancer-related pain and postoperative surgical pain. Among the study population, 10 patients were managed with epidural port systems, all of whom had a catheter duration exceeding 20 days. Procedural characteristics, including catheter insertion site, insertion level, and indication for catheter placement, are summarized in Table 1.

### 3.2. Comparison Between Patients with and Without Infection

Patients who developed infection had significantly longer catheter durations than those without infection. The median catheter duration was higher in the infection group, and this difference reached statistical significance (*p* = 0.001).

No statistically significant differences were observed between infected and non-infected patients with respect to catheter insertion site or insertion level. The distribution of diagnostic groups showed a higher proportion of cancer-related pain among patients with infection, although this difference did not reach statistical significance in univariable analysis. Detailed comparisons between patients with and without infection are presented in Table 2.

### 3.3. Multivariable Logistic Regression Analysis

Variables associated with infection in univariable analyses were entered into a multivariable logistic regression model. Catheter duration emerged as an independent risk factor for infection development. Each additional day of catheterization was associated with a significant increase in infection risk (odds ratio [OR] = 1.12; 95% CI: 1.06–1.19; *p* = 0.002).

When diagnostic groups were included in the model, cancer-related pain showed a tendency toward increased infection risk compared with other indications; however, this association did not reach statistical significance after adjustment. The results of the multivariable analysis are summarized in Table 3. Variables with *p* < 0.10 in univariable analysis were entered into the multivariable model.

### 3.4. Receiver Operating Characteristic (ROC) Analysis

**Figure 1:**
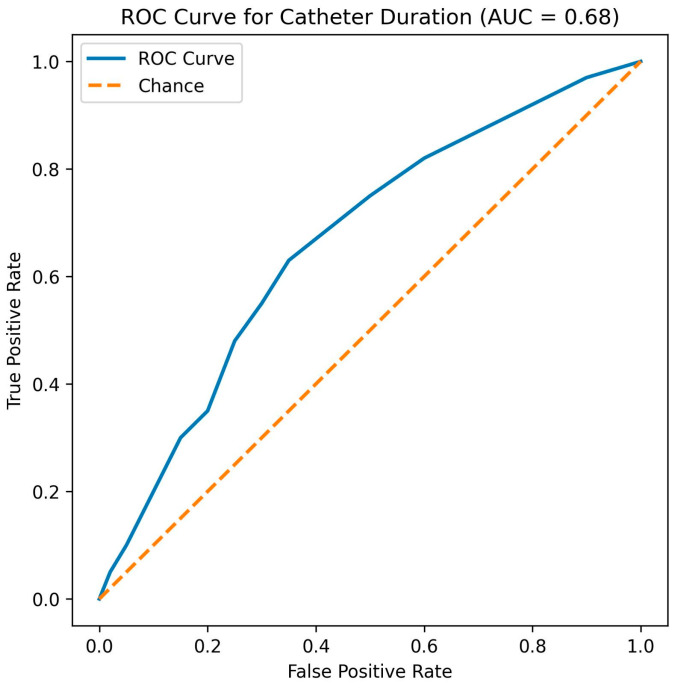
ROC curve for catheter duration. Receiver operating characteristic (ROC) curve showing the discriminatory ability of catheter duration for predicting epidural catheter- or port-related infection.

ROC curve analysis demonstrated that catheter duration had a moderate discriminatory ability for predicting infection development, with an area under the curve (AUC = 0.68) (95% CI: 0.59–0.76). The optimal cut-off value for catheter duration was identified as 7 days, yielding a sensitivity of 64.9% and a specificity of 67.0%**.** ROC curve characteristics and the optimal cut-off point are shown in Figure 1.

### 3.5. Microbiological Findings

Microbiological culture samples were obtained for all patients classified as having infection; however, the diagnosis of infection was based primarily on documented clinical findings, and culture results were used to identify microorganisms when bacterial growth was detected. The most frequently isolated pathogens were methicillin-resistant coagulase-negative staphylococci (MRCNS), accounting for 45.9% of infections. Other identified microorganisms included methicillin-resistant *Staphylococcus aureus* (MRSA) and Gram-negative bacteria, while the remaining cases consisted of heterogeneous pathogens. The distribution of microorganisms is detailed in Table 4. Antimicrobial susceptibility testing was performed for all culture-positive isolates, and antibiotic therapy was adjusted accordingly based on susceptibility results. None of the patients developed meningitis or sepsis, and all infections were successfully managed with antibiotic therapy.

## 4. Discussion

This retrospective observational study investigated infectious complications related to epidural catheter and epidural port applications in a large cohort followed by a tertiary pain clinic. The principal findings of the study were that catheter-related infection occurred in a clinically relevant proportion of patients, catheter duration was the most important independent risk factor for infection development, a dwell time of 7 days represented a meaningful threshold for increased risk, and methicillin-resistant coagulase-negative staphylococci constituted the predominant bacterial pathogens. These findings provide contemporary evidence to inform safer catheter management strategies in pain medicine practice [2].

Epidural catheter-related infection rates reported in the literature vary widely depending on the patient population, catheter duration, and clinical setting. In perioperative anesthesia populations where catheters are typically used for short durations, infection rates are generally reported to be below 1% [14]. In contrast, studies conducted in chronic pain or palliative care populations have described higher infection rates, largely due to prolonged catheter dwell times and repeated catheter manipulation [15,16]. The infection rate observed in the present cohort, therefore, reflects the specific characteristics of a tertiary pain clinic population, in which long-term neuraxial analgesia is frequently required.

The infection rate observed in the present cohort appears higher than that reported in many perioperative epidural catheter studies; however, this difference should be interpreted in the context of the study population and clinical setting [14,15,16]. Unlike short-term perioperative cohorts, our study reflects a tertiary pain clinic population in which catheter duration was substantially longer, repeated catheter access was more frequent, and prolonged neuraxial analgesia was often required. These features are likely to increase the risk of clinically apparent catheter-related infection and may partly explain the higher infection rate observed in the present study. In addition, all cases included as infections had documented local clinical findings, and colonization-only cases were excluded from the analysis after retrospective case review. Nevertheless, a degree of overestimation inherent to retrospective designs cannot be fully excluded.

The overall infection rate observed in the present cohort (10.5%) is consistent with infection rates reported in recent studies focusing on prolonged neuraxial catheter use outside the operating room setting [10]. Contemporary reviews and cohort studies published within the last decade have emphasized that infection rates in pain medicine populations tend to be higher than those reported in short-term perioperative anesthesia cohorts, largely due to extended catheter dwell times and patient-related risk factors [6,7,17]. More recent analyses have confirmed that infection risk remains a persistent concern in chronic pain populations, even with strict aseptic protocols [18].

In this study, epidural port systems were used in a limited number of patients requiring prolonged analgesic therapy, all with catheter durations exceeding 20 days. Infection occurred in 4 of the 10 port patients; however, none developed meningitis or sepsis, and all infections were successfully treated with antibiotic therapy. These findings indicate that although epidural ports are associated with longer indwelling times and repeated access, severe infectious complications can be minimized with appropriate monitoring and strict aseptic care. In this context, catheter duration remains the primary determinant of infection risk [10].

Among all evaluated variables, catheter duration emerged as the most robust and consistent predictor of infection [3]. This finding aligns closely with recent literature identifying prolonged catheterization as the primary modifiable determinant of neuraxial infection risk [7,19]. Large observational datasets and registry-based analyses published in the last five years have demonstrated a near-linear relationship between catheter dwell time and infection probability, reinforcing the time-dependent nature of this complication [20].

The biological plausibility of this association is well established. Prolonged catheter presence facilitates bacterial migration along the catheter tract and promotes biofilm formation, which remains resistant to host immune responses and antimicrobial therapy [12]. Although this mechanistic framework originates from earlier studies, it remains highly relevant and continues to underpin modern infection prevention strategies [21].

Biofilm formation on indwelling medical devices has been widely recognized as a key mechanism underlying device-related infections. Once bacteria adhere to catheter surfaces, they can form structured biofilms that protect microorganisms from host immune responses and antimicrobial therapy [22]. This process develops progressively over time, which may partly explain the time-dependent increase in infection risk observed with prolonged catheterization. In the context of neuraxial catheters, biofilm formation may initially present as bacterial colonization before evolving into clinically apparent infection, highlighting the importance of early detection and careful catheter monitoring.

The identification of a 7-day cut-off for catheter duration represents one of the most clinically actionable findings of this study. Recent investigations in both regional anesthesia and pain medicine have suggested that infection risk increases substantially after 5–7 days of catheterization, particularly in non-surgical pain populations [23]. Our findings support these observations and provide institution-specific data that may aid clinicians in balancing analgesic benefit against infection risk. ROC analysis was performed to provide a clinically applicable time-based threshold rather than to develop a predictive model. It is important to note that the moderate discriminatory performance observed in ROC analysis reflects the multifactorial nature of catheter-related infections. Nevertheless, the 7-day threshold may serve as a pragmatic trigger for intensified monitoring, prophylactic measures, or planned catheter removal.

Although patients with cancer-related pain demonstrated a trend toward higher infection rates, the diagnostic category did not remain an independent predictor after adjustment for catheter duration [24]. Recent studies have similarly suggested that procedural factors may outweigh patient diagnosis in determining infection risk, particularly when standardized insertion techniques and follow-up protocols are employed [25]. This finding underscores the importance of focusing on modifiable procedural variables rather than patient diagnosis alone.

The predominance of methicillin-resistant coagulase-negative staphylococci observed in this study is consistent with microbiological patterns reported in recent large-scale analyses of catheter-related infections [13]. Contemporary surveillance studies continue to identify skin-derived organisms as the leading causative pathogens, reflecting the critical role of insertion-site contamination despite advances in antiseptic techniques [26]. The identification of MRSA and Gram-negative organisms, although less frequent, highlights the potential severity of these infections and reinforces the need for early recognition and targeted antimicrobial therapy.

All patients with catheter-related infections were successfully treated with antibiotic therapy, and no cases of sepsis or infection-related mortality were observed. This finding should be interpreted in light of the study design, as patients with pre-existing systemic infection or sepsis were excluded.

From a practical standpoint, these findings emphasize that catheter duration should be a central consideration during treatment planning. In patients anticipated to require prolonged neuraxial analgesia, alternative strategies or scheduled reassessment may reduce infection risk [27]. Furthermore, structured follow-up within specialized pain clinics, as implemented in the present study, may facilitate early detection and timely intervention, thereby limiting morbidity. Nevertheless, due to the retrospective design, the absence of precise infection onset timing, and the lack of detailed patient-related variables such as comorbidities, immunosuppression status, and prior antibiotic exposure, a degree of uncertainty in temporal risk estimation and residual confounding cannot be fully excluded.

Future research should aim to further clarify the mechanisms and clinical determinants of epidural catheter-related infections in chronic pain populations. Prospective multicenter studies with standardized microbiological surveillance and precise documentation of infection onset may provide a more accurate understanding of time-dependent infection dynamics. Such data could contribute to the development of evidence-based guidelines for safer long-term neuraxial catheter management in pain medicine practice.

## 5. Limitations

Several limitations should be acknowledged. The retrospective design limits causal inference, and certain patient-related factors, such as detailed immunological status or peri-procedural antibiotic use, could not be fully assessed. Another limitation is the relatively small number of patients with epidural port systems, which limits the ability to draw firm conclusions regarding infection risk specifically associated with epidural ports. Because of the retrospective design of the study, blood culture results were not consistently available for all patients with suspected infection, which limited the possibility of evaluating microbiological concordance between catheter-related cultures and systemic samples. Because the exact timing of infection onset was not systematically recorded in the medical records, time-to-event analysis using Cox proportional hazards regression could not be performed. Additionally, the single-center nature of the study may restrict generalizability. Nevertheless, the relatively large sample size and contemporary clinical setting enhance the relevance of the findings.

## 6. Conclusions

In conclusion, epidural catheter and epidural port applications are associated with a measurable risk of infection in modern pain practice. Catheter duration remains the most important modifiable risk factor, with a clinically meaningful increase in infection risk beyond 7 days. These results support time-based risk stratification and may contribute to safer neuraxial catheter management strategies in pain clinics.

## Figures and Tables

**Table 1:** Patient and procedural characteristics (*n* = 352).

Variable	Value
Type of epidural system, *n* (%)	
Conventional epidural catheter	342 (97.2)
Epidural port system	10 (2.8)
Catheter duration (days), median (min–max)	5 (1–86)
Catheter placement level, *n* (%)	
Lumbar	264 (75.0)
Thoracic (T)	88 (25.0)
Indication for catheter placement, *n* (%)	
Cancer pain	118 (33.5)
Postoperative pain	35 (9.9)
Other	199 (56.5)

**Table 2:** Comparison of patients with and without epidural catheter- or port-related infection.

Variable	Infection (*n* = 37)	No Infection (*n* = 315)	*p* Value
Catheter duration (days), median (min–max)	9 (3–30)	5 (1–25)	0.001
Diagnosis group, *n* (%)			
Cancer-related pain	17 (45.9)	101 (32.1)	0.094
Postoperative surgical pain	5 (13.5)	30 (9.5)	
Other indications	15 (40.6)	184 (58.4)	
Catheter insertion site, *n* (%)			0.418
Insertion level, *n* (%)			0.507

**Table 3:** Multivariable logistic regression analysis of risk factors associated with epidural catheter- or port-related infection.

Variable	OR	95% CI	*p* Value
Catheter duration (per day)	1.12	1.06–1.19	0.002
Diagnosis group			
Cancer vs. Other	1.54	0.78–3.02	0.212
Postoperative surgery vs. Other	1.31	0.46–3.69	0.610

**Table 4:** Distribution of microorganisms isolated in patients with epidural catheter- or port-related infection (*n* = 37).

Microorganism	*n* (%)
Methicillin-resistant coagulase-negative staphylococci (MRCNS)	17 (45.9)
Methicillin-resistant *Staphylococcus aureus* (MRSA)	4 (10.8)
*Klebsiella pneumoniae*	3 (8.1)
Other microorganisms *	13 (35.1)

* Other microorganisms included *Enterococcus* spp., *Streptococcus* spp., *Enterobacter* spp., *Acinetobacter* spp., and other Gram-negative isolates identified in small numbers.

## Data Availability

The data that support the findings of this study are available upon request from the corresponding author. The data are not publicly available due to privacy or ethical restrictions.
